# Antioxidant Properties of two Edible Green Seaweeds From Northern Coasts of the Persian Gulf

**Published:** 2013-02-13

**Authors:** Massoumeh Farasat, Ramazan-Ali Khavari-Nejad, Seyed Mohammad Bagher Nabavi, Foroogh Namjooyan

**Affiliations:** 1 Department of Biology, Science and Research Branch, Islamic Azad University, Tehran, IR Iran; 2 Faculty of Marine Science, University of Marine Sciences and Technology, Khorramshahr, IR Iran; 3 Marine Pharmaceutical Research Center, Pharmacognosy Department, School of Pharmacy, Jundishapur University of Medical Sciences, Ahvaz, IR Iran

**Keywords:** Antioxidant Capacity, Total Phenolics, Flavonoid, DPPH

## Abstract

**Background:**

*Ulva* genus, an edible seaweed, and an important food source in many south-east Asian countries is also recognized by its synonymous name as *Enteromorpha*.

**Objectives:**

This study was carried out to evaluate antioxidant activity, contents of total phenolics, and flavonoids of methanolic extracts of edible green seaweeds including *Ulva clathrata* (Roth) *C. Agardh* and three samples of *Ulva prolifera* O.F.Müller grown at different parts of Bushehr Province along the northern coasts of the Persian Gulf.

**Materials and Methods:**

The seaweeds were collected from Bordekhoun, Northern Ouli, Taheri and Kangan coasts in December 2011. Methanolic extracts of the seaweeds were assessed for their antioxidant activity using DPPH (1,1-diphenyl-2-picrylhydrazyl) radical scavenging assay and was performed in a microplate reader. Total phenolics were determined by Folin-Ciocalteu reagent and flavonoid content was evaluated by colorimetric method.

**Results:**

All samples showed antioxidant activity to various degrees. *Ulva clathrata* exhibited a high DPPH radical scavenging activity with a low IC_50_ (the half-maximal inhibitory concentration) (0.715 ± 0.078 mg. mL^-1^). The highest phenolic content (4.468 ± 0.379 mg GAE g^-1^) (gallic acid equivalent) and flavonoid content (45.577 ± 0.949 mg RE g*-1*) (rutin equivalent) were also observed in *U .clathrata*. The phenolic and flavonoid contents showed positive correlations with the DPPH radical scavenging activity and negative correlations with IC_50_ (P < 0.01). Besides, Results showed that there was a positive correlation between total phenolics and flavonoid content of extracts (P < 0.01).

**Conclusions:**

Strong positive and significant correlations between DPPH radical scavenging and phenolic and flavonoid contents showed that, phenolic compounds, including flavonoids are the main contributors of antioxidant activity in these *Ulva* species and variations in phenolics and flavonoid contents of the seaweed extracts may be due to the variation in physicochemical parameters such as salinity amongst the selected stations.

## 1. Background

Reactive oxygen species (ROS) have been implicated in pathogenesis of many diseases, including cancer, mutagenesis, Alzimer’s disease, AIDS, etc. Many synthetic antioxidants are currently in use, nevertheless, there is a growing evidence of consumer preference for natural antioxidants because of their potentially lower toxicity (1).

According to the previous studies, terrestrial plants are rich sources of phytochemicals possessing important properties such as antioxidant activity. Many investigators have found several types of antioxidants from different parts of various plant species such as oilseeds, cereal crops, vegetables and spices (2).

Recently, polyphenolic compounds including flavonoids are known as safe and non-toxic antioxidants. Many studies have shown that a high dietary intake of natural phenolics is strongly associated with longer life expectancy, reduced risk of developing some chronic diseases, various types of cancer, diabetes, obesity, improved endothelial function and reduced blood pressure (3-5).

Phenolic compounds are commonly found in plants and seaweeds. Seaweeds are known to contain a wide variety of bioactive compounds, many of which have commercial applications in pharmaceutical, medical, cosmetic, food industries and agriculture (6). It has been observed that ROS production in algae is stimulated by various environmental stresses, such as high light levels, heavy metals, high salt concentrations, UV radiation etc. Algae generally has higher antioxidant activity due to higher contents of nonenzymatic antioxidant components, such as ascorbic acid, reduced glutathione, phenols and flavonoids (7). As a result, many marine bio-sources in the last decades have attracted attention in the search for natural bioactive compounds to develop new drugs and healthy foods. Compounds with antioxidant, antiviral, antifungal, antimicrobial, antitumor and anti-inflammatory activities have been found in brown, red and green algae (8). The antioxidant activity of several seaweeds has been reported (9). *Ulva* genus is an important food source in many south-east Asian countries which is also recognized by its synonymous name as *Enteromorpha*. However, research on the use of this green seaweed genus for food or the treatment of various diseases has received less attention in Iran.

## 2. Objectives

The antioxidant properties of *Ulva clathrata* and *U. prolifera* from Iran have not been previously published. The present study aimed to investigate the antioxidant capacity, total phenolics and flavonoids of these edible seaweeds from the northern coasts of the Persian Gulf for future applications in medicine, dietary supplements, cosmetics or food industries.

## 3. Materials and Methods

### 3.1. Chemicals

Ascorbic acid, Folin-ciocalteu reagent, Gallic acid and Methanol were purchased from Merck Company (Darmstadt, Germany). DPPH (1, 1-diphenyl-2-picrylhydrazyl) and Rutin were purchased from Sigma Chemical Co. (St. Louis, MO, USA). All the chemicals and reagents used were of analytical grade.

### 3.2. Collection and Preparing of Algal Extract

The seaweeds were collected at low tide time (according to the tide time table obtained from www.iranhydrography.org) along the northern coasts of the Persian Gulf from Bordekhoun, Northern Ouli, Taheri and Kangan in December 2011. The latitude and longitude of each sampling location was recorded by GPS tracking device. Once harvested, seaweeds were washed with fresh water to remove sands, salts and epiphytes, and then were air-dried at room temperature with good controlled air condition carefully. Voucher specimens were pressed and, stored in 5% formol for identification. Samples were observed under a light microscope for anatomical examination. The samples were identified according to the characteristics and identification keys in the taxonomic publications (10-14). Herbarium voucher specimens were kept in Jundishapur marine pharmaceutical research center (JMPRC) herbarium and samples were kept at -50º^C^ until experiments were processed and milled into powder before extraction.

Dried seaweed sample (200 mg) was extracted with 6 mL 80% methanol in an ultrasonic bath for 20 min, vortexed for 30 min then left to stand at room temperature for 48 h. The extract centrifuged at 1500g for 10 min and filtered through Watmann No.1 paper filter. The stock solutions of extracts were adjusted with 80% methanol to final concentration of 2 mg mL^-1^. Dilutions were made to obtain concentrations 1, 0.5 and 0.1 mg mL^-1^.

### 3.3. DPPH Free Radical Scavenging Activity

DPPH radical scavenging activity was determined according to the method of Zhang *et al*. (2007) with slight modifications (15). Briefly, 100 µl of each extract at various dilutions, were mixed with 100 µl of 0.16 mM DPPH methanolic solution. The mixture was vortexed for 1 min, kept for 30 min in dark and then, the absorbance was read at 517 nm in an automated microplate reader (Sunrise-Elisa Reader,Tecan ,Swiss). The antioxidant capacity was calculated using the following equation:

% Inhibition = (Acotrol - (Asample - Ablank) / Acotrol) × 100

Where the Acotrol is the absorbance of the control (DPPH without sample), the Asample is the absorbance of the test sample (the sample test and DPPH solution), and the Ablank is the absorbance of the sample blank (Sample without the DPPH solution). The half-maximal inhibitory concentration IC_50_ (the half-maximal inhibitory concentration) was calculated by linear regression analysis and expressed as mean of three determinations. Ascorbic acid was used as positive control.

### 3.4. Determination of Total Phenolic Compounds and Flavonoid Content

Total phenolic compounds (TPC) of algal extracts was determined by Folin-Ciocalteu reagent according to the method of Antolovich *et al*. (2002) (16) with minor modifications. In Brief, 20 µL of extracts were mixed with 100 µl of 1:10 Folin-Ciocalteu reagent followed by the addition of Na_2_CO_3_ (80 µL, 7.5%). The assay was carried out in microplate. After incubation at room temperature for 2 hours in dark, the absorbance at 600 nm was recorded. Gallic acid was used as the standard reference. TPC (total phenolic content) was expressed as mg gallic acid equivalents per gram of dried extract (mg GAE g^-1^) (gallic acid equivalent).

Flavonoid content (FC) (flavonoid content) of each extract was determined by following colorimetric method (17). Briefly, 20 µL of each extract was separately mixed with 20 µL of 10 % aluminium chloride, 20 µL of 1 M potassium acetate and 180 µL of distilled water, and left at room temperature for 30 min. The absorbance of the reaction was recorded at 415 nm. The calibration curve was prepared by rutin methanolic solutions at concentrations of 12.5 to 100 µg mL^-1^. FC was expressed as mg rutin equivalents per gram of dried extract (mg RE g^-1^) (rutin equivalent).

### 3.5. Statistics

Data were expressed as means ± standard errors of three replicate determinations. Statistical analyses were carried out using SPSS 16.0 for Windows. To determine whether there were any differences among the means, one way analysis of (ANOVA) and the Duncan’s new multiple range test were applied to the result at 0.05 level of significance (P < 0.05). The Pearson correlation analysis was performed between variables.

## 4. Results

### 4.1. DPPH Radical Scavenging Activity

During the study, *Ulva clathrata* and three samples of *Ulva prolifera* were collected from middle and lower intertidal zones of northern coasts of the Persian Gulf. The scientific names of seaweeds and their collection locations are listed in Table 1. All seaweed extracts showed antioxidant activity to various degrees ( Table 2 ). Lower IC_50_ value indicates higher antioxidant activity. As shown in Table 2, the IC_50_ of *Ulva clathrata* extract was significantly different compared with those of *Ulva prolifera* extracts (P < 0.05).The IC_50_ of *Ulva prolifera* extracts (S2-S4) were not significantly different and was higher in *Ulva prolifera* grown in Northern Ouli (S4). The IC_50_ of ascorbic acid as a standard antioxidant was evaluated 0.037 ± 0.018 mg mL^-1^ in this work and was significantly different in comparison to the seaweed extracts (P < 0.05). The scavenging effect of ascorbic acid ranged from 16.67 ± 2.98 % at concentration of 5 µg mL^-1^ to 90.34 ± .35 % at concentration of 100 µg mL^-1^.

**Figure 1:**
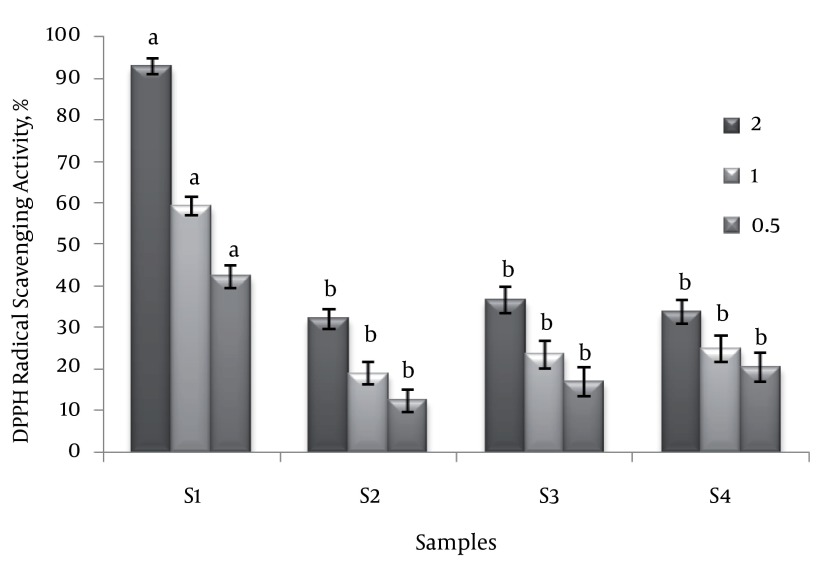
DPPH Radical Scavenging Activity of Extracts at Concentrations of 2, 1 and 0.5 mg mL^-1^ Each value is expressed as the mean ± SE (n = 3). Means with different letters (For each concentration level) are significantly different at P < 0.05

The scavenging effect of the tested extracts at concentration of 2 mg mL^-1^ on the DPPH radical decreased in the order of: S1 > S3 > S4 > S2, and were 92.7 ± 1.84, 36.59 ± 3.09, 33.82 ± 3.00 and 32.15 ± 2.45 % , respectively (Figure 1). It can be noticed that the DPPH radical scavenging values of all extracts were dose dependent in the range of the tested concentrations. However, the extract of *U. clathrata* was found to be the most potent scavenger in the tested algae. The DPPH radical scavenging activity of the *U. clathrata* extract in the range of the tested concentrations was significantly different in comparison to those of the *U. prolifera* extracts. The scavenging effect of the *U. clathrata* extract at concentration of 2 mg mL^-1^ was comparable to that of the positive control, ascorbic acid at concentration of 0.1 mg mL^-1^.

**Table 1.** The Seaweeds and Their Collection Information

ID code	Sample	Scientific name	Locality	Latitude, Longitude
**G111032**	**S1**	*Ulva clathrata (Roth) C. Agardh*	Bordekhoun	N2800306,E05122437
**G110831**	**S2**	*Ulva prolifera * *O.F. Müller*	Kangan	N2740024,E05219893
**G110731**	**S3**	*Ulva prolifera * *O.F. Müller*	Taheri	N2740040,E05219711
**G110932**	**S4**	*Ulva prolifera * *O.F. Müller*	Northern Ouli	N2750316,E0515308

**Table 2:** IC50, TPC and FC of the Seaweed Extracts

Sample	Species	IC_50_ (mg mL^-1^)	TPC(mg g^-1^)	FC(mg g^-1^)
**S1**	*Ulva clathrata*	0.715 ± 0.078 a	4.468 ± 0.379 a	45.577 ± 0.949 a
**S2**	*Ulva prolifera*	3.101 ± 0.107 b	1.371 ± 0.148 b	10.184 ± 1.044 b
**S3**	*Ulva prolifera*	3.026 ± 0.221 b	1.465 ± 0.068 b	9.370 ± 1.252 b
**S4**	*Ulva prolifera*	3.026 ± 0.221 c	1.449 ± 0.138 b	7.959 ± 0.769 b

Each value is expressed as the mean ± SE (n = 3)

^a, b, c^ Means with different letters are significantly different at P < 0.05

### 4.2. Total Phenolic Compounds and Flavonoid Content

Total phenolic content (TPC) and flavonoid content (FC) of the algal extracts are also presented in Table 2. The phenolic content of *U. clathrata* extract was significantly different compared with those of the other species (P < 0.05). In general, the higher total phenolic content resulted in higher antioxidant capacity. As shown in Table 2, the flavonoid content of algal extracts varied from 45.577 ± 0.949 (*Ulva clathrata*) to 7.959 ± 0.769 (*U. prolifera* grown in Northern Ouli) mg RE g^-1^. The flavonoid content of *U. clathrata* extract was significantly different (P < 0.05) as compared with the other species. The flavonoid contents of the *U. prolifera* samples (S2-S4) were not significantly different and were higher in S2 which was collected from Kangan.

The Pearson’s correlation coefficients between the variables are presented in Table 3. As shown in this table, there were strong positive and significant correlations between DPPH radical scavenging and contents of phenolics and flavonoids, and high negative correlations between IC_50_ and the variables and these correlations were stronger for flavonoid contents than the phenolic contents. Also, the results revealed that there was a strong positive correlation between flavonoids and total phenolics of the seaweeds extracts (P < 0.01).

**Table 3:** Pearson’s Correlation Coefficients Between the Variables

	TPC	FC	IC50
**TPC**	0.957 a		
**IC50**	-0.898 a	-0.957 a	
**DPPH radical scavenging activity**	0.959 a	0.985 a	-0.935 a

^a^ Correlation is significant at the 0.01 level (2-tailed)

## 5. Discussion

Due to the presence of different bioactive components with antioxidative potential in the crude extracts of the samples, many different methods have been used to investigate various sample extracts in recent years. In the current study, the DPPH radical scavenging method was used to evaluate the antioxidant capacity of the seaweed extracts, because the use of DPPH radical provides an easy, rapid and convenient method to evaluate the antioxidants and radical scavengers (18).

Many studies have been done to determine antioxidant capacity in *Ulva* species and some researchers have stated high scavenging activity for *Ulva* species. For instance, higher scavenging capability of polysaccharides obtained from *U. prolifera* extract than that of butylated hydroytoluene(BHT) to DPPH radical has been reported (65.2 % at the concentration of 0.5 mg mL^-1^) (19). Also, three edible species of *Ulva* including *U.compressa*, *U. linza* and *U. tubulosa* were assessed for evaluating their antioxidative activities and all the tested seaweeds exhibited high antioxidant activity in linoleic acid system and the best DPPH radical scavenging was observed in methanolic extract of *U. compressa* (IC50 =1.89 mg mL^-1^) (20). It has been shown that, chronic consumption of polysaccharides supplied by *Ulva* species; prevent the fall of antioxidant defences and the development of atherosclerosis in hamsters (21). Besides, Polysaccharides obtained from *U. lactuca* extract with potent hypocholesterolemic and antioxidant effects in experimentally-induced hypercholesterolemic animal model have been reported (22). In addition, a high value of astaxanthin (a naturally occurring carotenoid pigment and a powerful antioxidant) has been reported in *Ulva* species (23). Moreover, some researchers have isolated natural Ulvan, a group of sulfated heteropolysaccharides and its derivatives (24) and sesquiterpenoids (25) from *Ulva* species with high free radical scavenging properties.

In the current study, the antioxidant activity of *Ulva* species was in accordance with their amount of phenolic and flavonoid contents. Several reports have indicated a close relationship between total phenolic content and high antioxidant activity, and many researchers demonstrated that phenolic compounds were one of the most effective antioxidants in marine algae (26, 27).

The best-described property of almost every group of flavonoids is their capacity to act as antioxidants (28). A positive correlation was documented between antioxidation capabilities and total polyphenol contents for *Ulva prolifera*, but not with the contents of flavonoids (29). On contrary, Cho *et al* (30). (2011) (30) reported little correlation between antioxidant activities and total phenolic contents of the extracts of *Ulva prolifera*. They suggested that the strong antioxidant activity of the extracts from *U. prolifera* was because of a chlorophyll compound, pheophorbide rather than phenolic compounds. In the current study strong, positive correlations was found between total phenol and flavonoid contents and the antioxidant capacity. Similar observation has been reported by Chai and Wong (2012) (31). The current research findings were in agreement with the results of Bouba *et al.* (2010) which reported a positive correlation between total phenolics and flavonoids in extracts of twenty Cameroonian spices (32).

Despite the fact that, species were from the same collection season, however, contents of their flavonoids and total phenolics were different. Previous studies had found marked changes in the chemical constituents with change of seasons and environmental conditions (33, 34) .These variations may be due to the variation in physicochemical parameters such as salinity amongst the selected stations.

In the current study, *U. clathrata* were collected from middle intertidal zone where the seaweeds are exposed to UV radiation for several hours in a day. *U. prolifera* samples were collected from lower intertidal zones. Prolonged seaweed exposure to solar UV radiation may result in producing bioactive compounds such as phenolics and flavonoids and may be an explanation of higher antioxidant capacity of *Ulva clathrata* in comparison with those of the other tested species.
